# Synergist for antitumor therapy: Astragalus polysaccharides acting on immune microenvironment

**DOI:** 10.1007/s12672-023-00798-w

**Published:** 2023-09-24

**Authors:** Qian Xu, Wen Cheng, Jinrui Wei, Yan Ou, Xian Xiao, Yingjie Jia

**Affiliations:** 1 Department of Oncology, First Teaching Hospital of Tianjin University of Traditional Chinese Medicine, Tianjin, China; 2 National Clinical Research Center for Chinese Medicine Acupuncture and Moxibustion, Tianjin, China

**Keywords:** Astragalus polysaccharides, Immune microenvironment, Antitumor, Immunotherapy, Digestive tract tumors

## Abstract

Various new treatments are emerging constantly in anti-tumor therapies, including chemotherapy, immunotherapy, and targeted therapy. However, the efficacy is still not satisfactory. Astragalus polysaccharide is an important bioactive component derived from the dry root of *Radix astragali*. Studies found that astragalus polysaccharides have gained great significance in increasing the sensitivity of anti-tumor treatment, reducing the side effects of anti-tumor treatment, reversing the drug resistance of anti-tumor drugs, etc. In this review, we focused on the role of astragalus polysaccharides in tumor immune microenvironment. We reviewed the immunomodulatory effect of astragalus polysaccharides on macrophages, dendritic cells, natural killer cells, T lymphocytes, and B lymphocytes. We found that astragalus polysaccharides can promote the activities of macrophages, dendritic cells, natural killer cells, T lymphocytes, and B lymphocytes and induce the expression of a variety of cytokines and chemokines. Furthermore, we summarized the clinical applications of astragalus polysaccharides in patients with digestive tract tumors. We summarized the effective mechanism of astragalus polysaccharides on digestive tract tumors, including apoptosis induction, proliferation inhibition, immunoactivity regulation, enhancement of the anticancer effect and chemosensitivity. Therefore, in view of the multiple functions of astragalus polysaccharides in tumor immune microenvironment and its clinical efficacy, the combination of astragalus polysaccharides with antitumor therapy such as immunotherapy may provide new sparks to the bottleneck of current treatment methods.

## Introduction

As one of traditional Chinese herbal medicine, *Radix astragali* has a long history of use in medicine. Many studies have discovered that *radix astragali* has a wide range of biological activities, such as anti-aging, anti-tumor, antioxidant, immunomodulation, and anti-inflammation. Thus, it is widely used in the treatment of cardiovascular diseases, diabetes mellitus, cancers, and other diseases [1–4]. Thereinto, *radix astragali* has complex chemical ingredients, the major components of which are polysaccharides (APS), flavonoids, and astragaloside IV(AS-IV) [2, 5, 6]. Amongst them, astragalus polysaccharides are the vital natural active component derived from *radix astragali*, the immunomodulatory nature of which is the most significant [7–9]. A large number of in vitro and in vivo studies have confirmed that astragalus polysaccharides can regulate the immune system. According to research, APS is an excellent immunopotentiator of both humoral and cellular immunity [10]. APS accelerates the maturation of DCs, improves their capacity for antigen presentation, and decreases their endocytic activity. Additionally, DCs could differentiate as a result of APS, which would then activate T cells [11]. APS is able to control T cell immunity. Through binding to anti-Toll-like receptor 4 (TLR4) on Tregs, APS might limit CD4^+^CD25^+^ Treg activity and cause the transition from T helper 2 cell (Th2) to T helper 1 cell (Th1) by engaging CD4^+^ T cells [12, 13]. Also, APS can activate B cells through membrane Ig in a way that is independent of TLR4 [14].

The tumor immune microenvironment (TIME) refers to the environment created by immune cells and their products in tumor tissues. It has been shown that TIME is directly associated to the development, growth, and spread of tumors as well as to the clinical prognosis of tumor patients [15, 16]. At present, there are more and more studies on the tumor immune microenvironment in clinic. For example, the study in gastric adenocarcinoma found that by modifying the innate as well as adaptive immune responses, we can influence the development of epithelial-derived gastric adenocarcinoma (GAC) by generating the immunosuppressive surroundings [17]. In addition, we also noticed that bidirectional interactions between gastric cancer stem cells (GCSCs) and immune cells in gastric cancer, about how cancer stem cells induce the reprogramming of leukocytes. And this could lead to immune cells that are pro-tumorigenic, orchestrating metastasis, chemoresistance, tumorigenicity, and even a rise in cancer cells with stem-like features. This might inspire fresh thought for immunotherapy targeting GCSC-related markers [18–20]. At present, immunotherapy has significantly outperformed traditional chemotherapy and radiotherapy in the treatment of cancers. Nevertheless, the immunosuppressive characteristics in tumor microenvironment (TME) cause difficulties in clinical efficacy and have severe side effects. Stimulating the TIME may be a critical step in improving the efficacy of current immunotherapies. According to the growing evidence, the effectiveness of immunotherapy can be multiplied by affecting the tumor immunosuppressive microenvironment [21–25]. Therefore, in-depth understanding the role of astragalus polysaccharides and tumor immune microenvironment is necessary to develop and optimize novel and effective cancer immunotherapies.

Digestive tract tumors are one of the most common malignant tumors globally, which have a high incidence and mortality rate [26]. The current treatment options of these tumors roughly include surgery, chemotherapy and radiotherapy, which are the first line treatments for advanced tumors that only prolong the survival of patients. However, tumors quickly develop strong resistance to chemotherapy medicines, while there is a high rate of recurrence and metastasis, and patients have a poor prognosis overall and serious adverse reactions [27]. Currently, immunotherapy may be one of the effective therapies to overcome the difficulties [28]. APS, a water-soluble heteropolysaccharide, stimulates and controls the immune system. The anticancer effect of APS may be enhanced by the promotion of the activities of macrophages, natural killer cells, dendritic cells, T lymphocytes, and B lymphocytes as well as the induction of a number of cytokines and chemokines [7, 29, 30]. With a focus on the application in the immunotherapy of digestive tract tumors, this review presents the research advances of APS in TIME.

## Regulation effect of APS on immunity

### Tumor-associated macrophages

Macrophages are main cells in the innate immune system, as well as the capital component of the mononuclear phagocyte system (MPS) [31]. Macrophages that appear in the TME are defined as tumor-associated macrophages (TAMs), which are widely present in various tumors [32]. While TAMs are the most infiltrated immune cells in the TME, they have been thoroughly investigated for their pro-tumoral actions, including tumor initiation, angiogenesis, metastasis, drug resistance, and antitumor immunosuppression [33–37]. It has been suggested that functional diversity of macrophages is relevant to their plasticity, and that molecules in TIME may adjust their functional phenotypes. Depending on the activation state, TAMs have a dual role on cancer with classically activated (M1) and alternatively activated (M2) cells commonly acting antitumor and protumor functions [38, 39]. M1 type macrophages have anti-tumor effects. Based on studies, M1 macrophages can destroy and clear tumor cells, which is consistent with their natural function to remove foreign objects. By activating pro-immunostimulating leukocytes and swallowing tumor cells, the M1 macrophages stimulate the cytokine production in the TME and promote tumor cell destruction [32]. M2 macrophages can promote tumor cell proliferation and invasion. There are studies found that M2 macrophages may contribute to basement membrane breakdown, deposition, angiogenesis, leukocyte recruitment, and overall immune suppression, and all of them could accelerate tumor development in both primary and metastatic sections [40–42]. Based on studies, M2 phenotype, which presents immunosuppression and promotes tumor growth, is dominant in TAMs. Therefore, the potential anticancer immunotherapy concentrates on M2 phenotype by eliminating them in the TME or converting M2 phenotype into M1 phenotype, which increases their cytotoxicity directly and indirectly prompts cytotoxic T cells to kill tumor cells [42–46]. Relevant studies have suggested that APS induce the polarization of macrophage to M1 phenotype by the Notch signaling pathway, which may enhance tumor killing and suppress tumor growth [47]. An experimental study in lung cancer found that APS can regulate the M1/M2 macrophage pool, facilitate DC maturation and enhance the anticancer effect of traditional chemotherapy drugs [48]. The research on breast cancer cell has demonstrated that APS can trigger the release of NO and tumor necrosis factor (TNF) from macrophages, possibly by activating of the TLR4-mediated MyD88-dependent signaling pathway, which directly blocks tumor growth [49–51]. The cell cycle arrest (G2 phase) and cell apoptosis via the mitochondrial apoptosis pathway induced by APS-mediated macrophages was found to dramatically restrict the development of 4T1 cells [52]. An animal experiment has shown that astragalus membranaceus polysaccharides (AMP) (100 and 400 mg/kg) were able to successfully limit the solid tumor growth in BALB/c mice grafted by H22 hepatocarcinoma. Moreover, AMP could increase the secretion of interleukin-2 (IL-2), IL-12 and TNF-α and lowered interleukin-10 (IL-10) level in serum [53]. In conclusion, APS can exert anti-tumor effects through influencing the polarization of TAMs in the immune microenvironment and induce the expression of a variety of cytokines, thus laying the foundation for further exploration of APS as a synergistic agent for immunotherapy.

### Dendritic cells

Tumor-associated antigens (TAAs) need to be transferred to lymphoid organs and delivered to T cells by antigen-presenting cells (APCs) in order to trigger effective immune responses. The primary professional APCs are macrophages, dendritic cells (DCs) and B cells. DCs have special capabilities and are more effective than macrophages and B cells at stimulating T cells for migration [54–56]. DCs continuously investigate peripheral tissue with dendrites, capture and process antigens, migrate towards lymphoid organs, and present antigens to T cells, which will directly lead to the T cell activation and polarization [57–61]. Furthermore, the diverse phenotypic and functional heterogeneity of DCs indicate their high plasticity and capacity to modify the acquired immune response based on TIME [62]. DCs have been shown in mouse cancer models to be able to collect tumor antigens secreted by tumor cells, whether they are still alive or have dead, and cross-present these antigens to T lymphocytes in lymph nodes. As a result, tumor specific cytotoxic T lymphocytes (CTLs) are generated, contributing to the tumor rejection [63, 64]. There are studies that have shown DCs in TIME can significantly impact the functions of antitumor T cells [65–68]. For example, the marginating DCs in TME can cross-present tumor antigens and continuously engage tumor specific T cells [69]. DCs in tumor-associated tertiary lymphoid structures signal Th1 cytotoxic immune environment and advance a protective T-cell-mediated immune response against tumors [70]. DCs could benefit from APS in a synergistic way. According to the research, APS can accelerate the mature of DCs. In order to have a better anticancer effect, APS may increase the expression of the surface molecules CD80 and CD86, accelerate DCs maturation, and activate CTLs [71]. In mice given the HBV-DNA vaccination, APS (500 ug/mouse) as an adjuvant could promote DCs maturation and increase the expression of major histocompatibility complex I (MHC I), major histocompatibility complex II (MHC II), CD40, CD80, and CD86 [10]. By improving the expression of CD40, CD80, CD86, and MHC II as well as promoting the generation of NO, the pure polysaccharide APSII (1.67–45 ug/ml) may also be able to speed up the DCs mature [72]. Astragalus mongholicus polysaccharides (ASP) could increase the expression of CD11c and MHC class II molecules on DC surface and the secretion of IL-12, priming a strong stimulation of T lymphocytes growth and differentiation [11]. ASPC (3 mg/kg) might increase the number of CD80, CD103, and CD86 as well as promote the functional maturation of DCs in non-small cell lung cancer-bearing mice. This would improve the anticancer immune response carried out by T cells [48]. In addition, via improving the expression of MHC II, CD80, and CD86 on the surface of DCs, APS might stimulate the activation of DCs, which could strengthen the interactions between DCs and T cells [73]. In the study, APS can stimulate the differentiation of naturally IL-12 producing DCs, which can activate T lymphocyte immunological activity and transform Th2 to Th1 cells [74]. In summary, APS can strengthen the T cell-mediated antitumor immune responses by activating the differentiation and maturation of DCs. This may provide a new helper for tumor immunotherapy.

### Natural killer cells

The antiviral and anticancer features of natural killer (NK) cells make them significant effector lymphocytes [75, 76]. About 15% lymphocytes in circulation are NK cells, which are primarily found in the peripheral blood but also appear in a variety of organs such as the liver, lung, kidney and bone marrow [77, 78]. NK cells play a significant role in antitumor immunity by direct cytolysis and IFN-γ production as their cytotoxic effect is free of antigen pre-sensitization and there is no MHC restriction [79, 80]. NK cells are also effective in eliminating metastatic cancer cells and cancer stem cells [81–84]. So far, conventional tumor treatments can induce stress responses in tumor cells, which help NK cells identify them. For example, several chemotherapy drugs can increase the NK cell-activating ligands on tumor cells, thus increasing their sensitivity to NK cell-mediated lysis [85–87]. Meanwhile, several researches have showed that APS can influence the activity of NK cells and the expression of immune components like IFN-γ, TNF-α, granzyme-B and perforin, which might strengthen antitumor effect [29]. The mechanisms that APS activates NK cells were further studied. In H22 tumor-bearing mice, researchers found that the impaired signal transduction may lead to low response of NK cells, but APS can change the response state of NK cells in tumor cells. [88]. Experiments in vitro have demonstrated that the water-soluble polysaccharide AMP and its derivatives can use pro-inflammatory mediators and cytokines by NF-κB and mitogen-activated protein kinases (MAPKs) signaling pathways to stimulate the NK cells [89]. The intranasal treatment of APS increased the number of CD11c^+^ DCs in the mesenteric lymph nodes (mLN), which further activated NK cells and T cells. Thus, APS could be applied as an adjuvant to enhance the antitumor effect of immune checkpoint inhibitors [90]. In short, APS can affect antitumor efficacy of the drugs through regulating the activity of NK cells and increasing the expression of immune factors.

### T and B lymphocytes

It takes a longer time period for adaptive immune system to activate, which is related to activation of T and B lymphocytes (T and B cells), with huge specificity towards its targets and immunological memory. On the contrary, the innate immune system is activated quickly and nonspecifically in response to foreign pathogens or damaged self [91, 92].

**Fig. 1:**
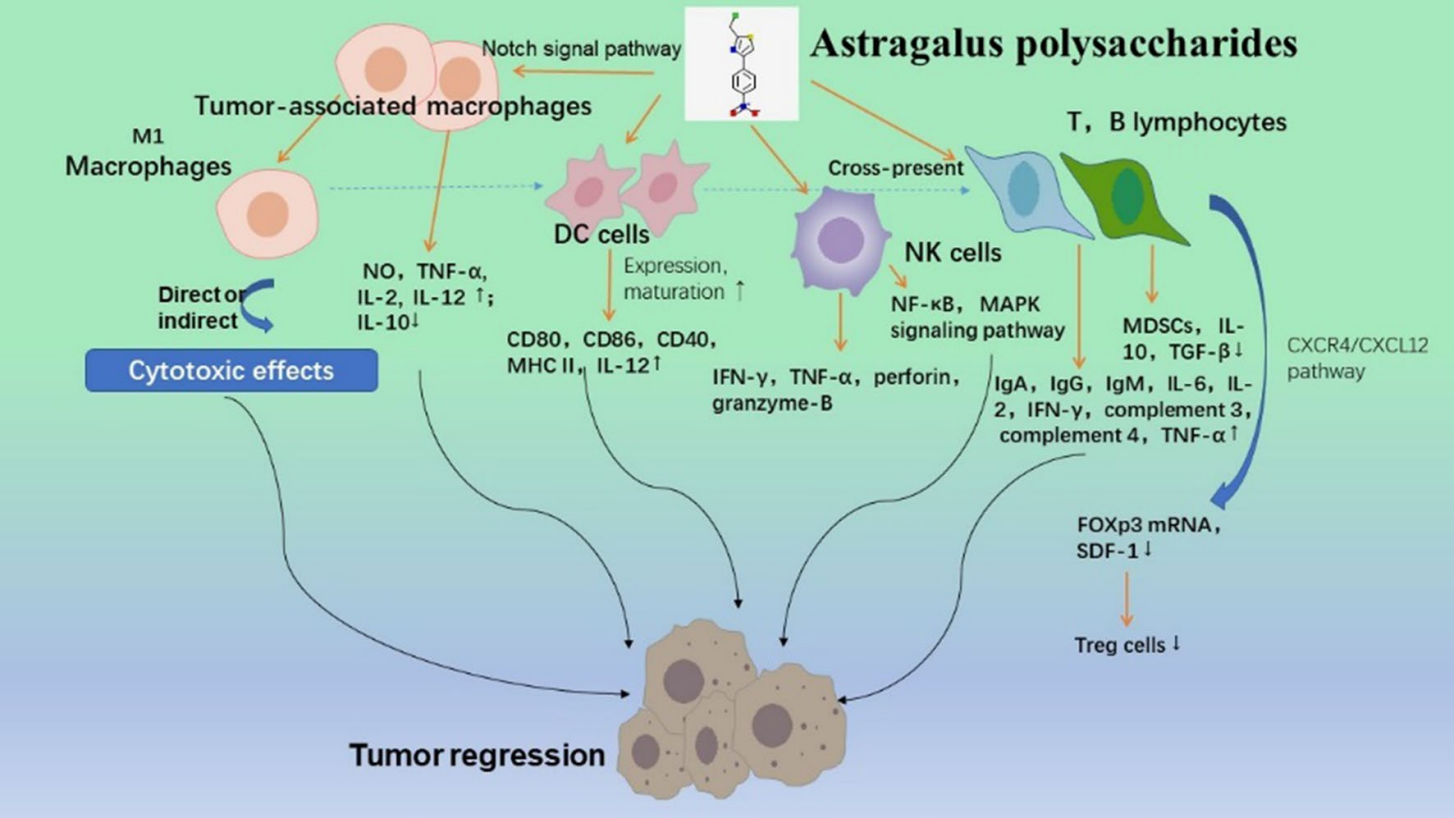
Astragalus polysaccharides acting on immune microenvironment

There are two crucial lymphocytes called T and B lymphocytes in the human body. Tumor-infiltrating B cells can produce antitumor effects not only by the production of tumor-specific antibodies and stimulation of T cell responses, but also by sustaining the tertiary lymphoid structure. [93]. Cytotoxic intra-tumoral CD4^+^ T cells have been found to kill cancer cells directly in preclinical and clinical trials. [94]. The CD4^+^ T helper cells and CD8^+^ cytotoxic T lymphocytes are two different subgroups of T cells divided on their function [95]. Three mechanisms that induce apoptosis in target cells include the secretion of proinflammatory cytokines, the interaction of the Fas ligand and Fas receptor, and the release of cytolytic granules containing perforin [96]. By regulating the activity of other immune cells like macrophages, neutrophils, B cells and CTLs, CD4^+^ T cells indirectly contribute in the removal of infections [97, 98]. By stimulating the proliferation of T and B cells as well as increasing the production of IgA, IgG, IgM, IFN-γ, interleukin-2 (IL-2), interleukin-6 (IL-6), complement 3, complement 4 and TNF-α, ASPC (8 mg/kg) was shown to improve immunity in cyclophosphamide-induced immunosuppressive mice in vivo [99]. Based on studies, APS could influence the growth of tumors in melanoma-bearing mice. It might decrease the quantity of myeloid-derived suppressor cells (MDSCs) and the expression of the cytokines IL-10 and TGF-β and the MDSC-related molecule Arg-1, which allowed CD8^+^ T cells to kill tumor cells more effectively [100]. Through activating antitumor immune cells and regulating the percentages of CD3^+^, CD4^+^, CD8^+^ T cells and CD19 B cells in tumor-bearing mice, APS have been found to be practical as a supplement for immune enhancement that can promote anaerobic metabolism of the TME and cell apoptosis [101]. Selenium-containing polysaccharides from the roots of astragalus membranaceus reduced CD4^+^ T cell apoptosis and serum cytokine dysregulation caused by tumor transplantation, which promoted the cytotoxic activities of NK cells and CD8^+^ T cells [102]. CD4^+^CD25^+^ Treg cell is known as a kind of T cells with immunosuppressive effect that can prevent the activation and proliferation of T cells [103, 104]. In the human hepatocellular carcinoma, APS can relieve the immune-suppressive effects of Treg cells by restoring the balance of cytokines in the TME, suppressing the expression of FOXp3 mRNA or inhibiting Treg cell migration by blocking SDF-1 and its receptor via the CXCR4/CXCL12 pathway [105]. The experiment has shown that APS could stimulate the proliferation of B cells which extracted from mouse spleen and cultured in vitro, but it is not sensitive to the proliferation of T cells [106]. The joint application of Chinese herbal extracts demonstrated additional benefits. In mice with lung cancer, the combination of the polysaccharopeptide (PSP) and APS dramatically improved the level of WBC, thymus index, spleen index, CD4/CD8 ratio, TNF, IFN-γ, IL-2 and interleukin-17 (IL-17), which indicated their immunomodulatory effects and antitumor activity [107]. In summary, by accelerating the proliferation of T and B cells, increasing the release of associated cytokines and suppressing the function of Treg cells, APS can improve immunosuppression and strengthen tumor immunity (Fig. 1).

## Application of Astragalus polysaccharides combined with immunotherapy

Currently, cancer immunotherapies approved by Food and Drug Administration (FDA) roughly included interferon, interleukin-2, dendritic cell vaccine, chimeric antigen receptor-T cells, anti-cytotoxic T lymphocyte antigen-4, anti-programmed cell death protein 1 (PD-1)/PD-L1 monoclonal antibodies, and so on [12]. APS has been demonstrated to improve the effect of immunotherapy in vivo and in vitro experiments [108, 109]. For example, by the AKT/mTOR/p70S6K pathway, PG2 can decrease the expression of PD-L1 on the cell surface, which may improve the efficacy of chemotherapy, and also decrease the expression of indoleamine 2, 3-dioxygenase 1 in tumor cells [109]. In an animal experiment on mice, APS can maintain an effective dose of anti-PD-1 antibodies in the body and can slow the tumor progression and genesis by boosting the activity of T cells. These findings may indicate that APS can enhance immune regulation or can be used as a supplement to therapy [110]. The cell experiment in vitro found that an appropriate dose of APS could upregulate the high expression of HLA-DR, CD86 and other co-stimulatory molecules related to antigen presentation on the surface of DC membrane, which had a significant effect on promoting DC differentiation and maturation, and increased the immune activity of DC [111]. By synergizing the cytotoxicity of cytokine-induced killer cells, APS can have cytotoxic effects on tumor cells. These are the same applied in lung cancer, cervical cancer, ovarian cancer and many other tumors [112–114]. To sum up, it is expected that APS will play an important role in the immunotherapy in the future.

## Application of APS in gastrointestinal tumors

### Application of APS in gastric cancer

Apoptosis induction: It has been shown that APS4 can induce MGC-803 cells apoptosis by promoting poly-ADP-ribose polymerase (PARP) cleavage and activating the expression of caspase 9/3, which result in accumulating intracellular ROS, releasing cytochrome c, increasing the expression of Bax and decreasing the expression of Bcl-2 [115]. With increasing the expression of caspase 3 that caused DNA fragmentation and promoting the expression of tumor suppressor genes via the MAPK signaling pathway, APS caused both the apoptosis of SGC-7901 cells and adriamycin resistant SGC-7901 cells [116]. Proliferation inhibition: SC-B, components of which are schisandrin B, aloe-emodin and APS, can restrict the proliferation and abnormal mitosis of human gastric cancer SCG-7901 cells in vitro. The inhibitory effect that leads to the cell cycle arrest of gastric cancer cells may be related to the decreased expression of cyclin D1 mRNA [117]. Enhancement of the anticancer effect of anticancer medicines: APS increased the anticancer effects of apatinib on gastric cancer AGS cells by lowering the levels of phosphorylated AKT and MMP-9 in the AKT signaling pathway [118]. Improvement of clinical efficacy and relief of chemotherapy adverse reactions: For patients with gastric cancer, the combination of the APS injection and the FOLFOX regimen (which consists of oxaliplatin, calcium folinate and 5 fluorouracil) seemed to be the most effective in terms of clinical efficacy as well as in relieving adverse reactions, particularly in leucopenia and gastrointestinal reaction [119].

### Application of APS in colorectal cancer

Proliferation inhibition: PG2 is a polysaccharide, isolated from the *radix astragali*, that suppresses the production and function of PD-L1 in tumors by the AKT/mTOR/p70S6K signaling pathway. With the combination of cisplatin and PG2, the growth of the mouse breast tumor and colorectal tumor was dramatically slowed [109]. Immunoactivity regulation: Effective antibodies, like anti-PD1 and anti-VEGF, can be generated by using APS that induces somatic mutation reaction in vivo. That may further enhance the antitumor immunity [110, 120].

### Application of APS in liver cancer

**Fig. 2:**
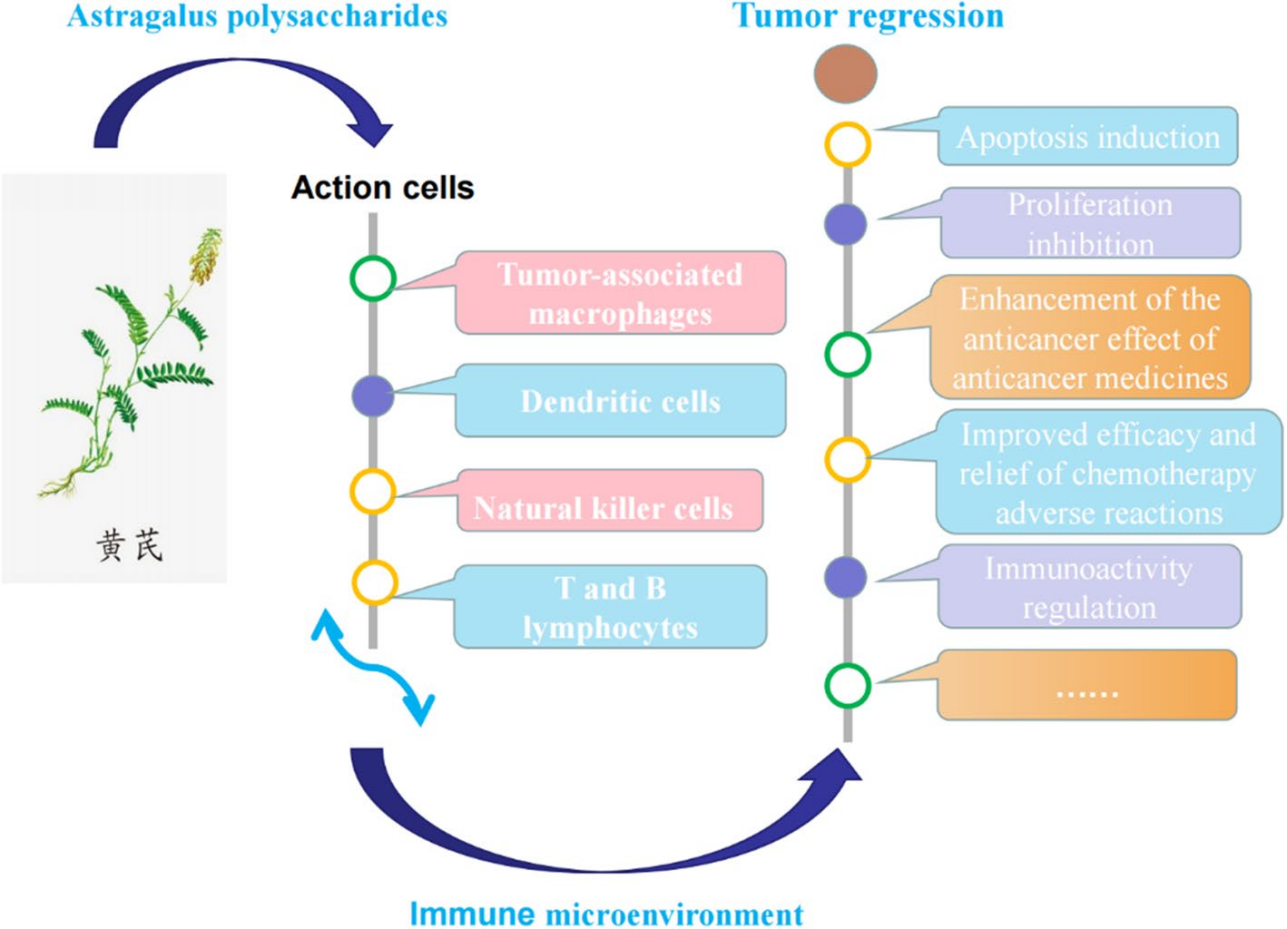
APS in immunotherapy of gastrointestinal tumors

Enhancement of chemosensitivity: APS can improve the chemosensitivity of Adriamycin-resistant H22 hepatoma cells. This may be related to the down-regulation of MDR1 mRNA, the inhibition of P-GP efflux pump function, which would lower the expression of the MDR1 protein [121]. Furthermore, by activating the JNK pathway, APS could enhance the sensitivity of SKOV3 cells to cisplatin, which is related to the apoptosis-related genes [122]. Immunoactivity regulation: To prevent the immunosuppressive effect of Treg cells, APS may restore the balance of cytokines in the TME and reduce the production of FOXp3 mRNA, which may enhance the antitumor effects of the immunotherapy-based methods [105]. In addition, by the miR-133a-3p/MSN axis, APS may also decrease the PD-L1-mediated immunosuppression, which leads to an anticancer effect [123]. Experiments of the hepatocellular carcinoma H22-bearing mice have showed that by raising the spleen and thymus indexes as well as increasing the cytokines like IL-2, IL-6, and TNF-α in serum, APS can affect immune-regulating features in tumor. In addition, APS increased the expression of the proteins Bax and decreased the expression of Bcl-2, which are related to the cell survival or death [53, 124, 125]. Relief of chemotherapy adverse reactions: APS can reduce the liver damage brought by cantharidin, which is involved in regulating bile acid biosynthesis and glycerophospholipid metabolism [126]. Additionally, APS is also effective in relieving the hepatotoxicity in mice that is caused by common chemotherapy drugs, such as cyclophosphamide, docetaxel and epirubicin [127] (Fig. 2 and Table 1).

**Table 1:** Related pharmacological activities or mechanisms of APS

Tumor	Features	Pathways or cells	Details	References
Gastric tumor	Apoptosis induction	Caspase-9/-3, ROS,cytochrome c,Bax,Bcl-2	Induce the apoptosis of MGC-803 cells by activating the expression of caspase-9/-3 and promoting PARP cleavage through intracellular ROS accumulation, the loss of mitochondrial membrane potential, the release of cytochrome c, the increased expression of Bax, and the reduced expression of Bcl-2	[115]
		MAPK signaling pathway, caspase-3, SEMA3F, P21W AF1/CIP1, FBXW7	Induce the apoptosis of SGC-7901 cells by enhancing the expression of caspase-3 and activating the MAPK signaling pathway	[116]
	Proliferation inhibition	Cyclin D1 mRNA	Inhibit the proliferation and aberrant mitosis of SCG-7901 cells through the down-regulation of cyclin D1 mRNA expression	[117]
	Enhancement of the anticancer effect	AKT signaling pathway, p-AKT, MMP-9	Inhibit of AKT signaling pathway and decrease the expression of phosphorylated AKT (p-AKT) and MMP-9 expression	[118]
Colorectal tumor	Proliferation inhibition	AKT/mTOR/p70S6K signaling pathway, PD-L1	Inhibit tumor PD-L1 through modulating the AKT/mTOR/p70S6K signaling pathway	[109]
	Immunoactivity regulation	Anti-VEGF	Induce somatic mutation reaction in vivo, and increase anti-VEGF	[120]
		Anti-PD1	Elevate cytokine and anti-PD-1 antibody titers	[110]
Liver tumor	Enhancement of chemosensitivity	MDR1 mRNA, P-GP efflux pump	Downregulate MDR1 mRNA expression and inhibit P-GP efflux pump function	[121]
		JNK pathway	Increase the sensitivity of SKOV3 cells to cisplatin by activating the JNK pathway	[122]
	Immunoactivity regulation	FOXp3 mRNA, Treg cells	Suppress the expression of FOXp3 mRNA to inhibit the immune suppressive effects of Treg cells	[105]
		MiR-133a-3p/MSN axis, PD-L1	Attenuate PD-L1-mediated immunosuppression via miR-133a-3p/MSN axis	[123]
		IL-2, IL-6, TNF-α,Bax protein,Bcl-2	Increase the spleen and thymus indexes, and IL-2, IL-6, and TNF-α, and Bax protein expression and decrease Bcl-2 protein expression	[53, 124, 125]

## Summary and outlook

At present, the popular tumor treatments are composed of radiotherapy, chemotherapy and surgery while immunotherapy and targeted therapy also show great development prospects. Immunotherapy in tumor mainly includes immune checkpoint inhibitors, cancer vaccines, adoptive cellular therapy, NK cell and CAR T cell therapy, etc. [128–131]. Yet, there is still a lot to be done for research.

APS and its related preparations have long been used clinically. Among them, APS is the most eye-catching in terms of immune regulation. APS can regulate the activity of diverse immune cells and further improve the TIME. Macrophages, NK cells, DCs, T cells and B cells are some of these immune cells. Moreover, APS can stimulate the production of various cytokines and chemokines generated by these immune cells, which strengthen the immunological response. Right now, researches on using APS to enhance immunomodulation or to be one complement of the treatment are in progress. We currently have a limited knowledge of the effects and the mechanisms that APS works in anti-tumor therapy. We need to deeper explore the mechanism of action of APS and related targets. However, we also require more explanation of its principle in antiaging, antitumor, antifibrosis, antibacterial and antiviral. APS is used in improving the immune functions of patients treated by chemical therapy or radiation therapy. One of the important factors in the prevention and treatment of diseases is the dose–response relationship [11]. Thus, more researches are essential to accurately control the dosage of APS that will make the most of its benefits. Hopefully, APS will be a strong ally in tumor immunotherapy with the advancement of clinical trials and combination medication researches.

## Data Availability

Not applicable.
